# A Novel Potassium Channel in Photosynthetic Cyanobacteria

**DOI:** 10.1371/journal.pone.0010118

**Published:** 2010-04-12

**Authors:** Manuela Zanetti, Enrico Teardo, Nicoletta La Rocca, Lalu Zulkifli, Vanessa Checchetto, Toshiaki Shijuku, Yuki Sato, Giorgio Mario Giacometti, Noboyuki Uozumi, Elisabetta Bergantino, Ildikò Szabò

**Affiliations:** 1 Department of Biology, University of Padova, Padova, Italy; 2 Department of Biomolecular Engineering, Graduate School of Engineering, Tohoku University, Sendai, Japan; Cairo University, Egypt

## Abstract

Elucidation of the structure-function relationship of a small number of prokaryotic ion channels characterized so far greatly contributed to our knowledge on basic mechanisms of ion conduction. We identified a new potassium channel (SynK) in the genome of the cyanobacterium *Synechocystis* sp. PCC6803, a photosynthetic model organism. SynK, when expressed in a K^+^-uptake-system deficient *E.coli* strain, was able to recover growth of these organisms. The protein functions as a potassium selective ion channel when expressed in Chinese Hamster Ovary cells. The location of SynK in cyanobacteria in both thylakoid and plasmamembranes was revealed by immunogold electron microscopy and Western blotting of isolated membrane fractions. SynK seems to be conserved during evolution, giving rise to a TPK (two-pore K^+^ channel) family member which is shown here to be located in the thylakoid membrane of *Arabidopsis*. Our work characterizes a novel cyanobacterial potassium channel and indicates the molecular nature of the first higher plant thylakoid cation channel, opening the way to functional studies.

## Introduction

Cyanobacteria, the first organisms capable of performing oxygenic photosynthesis during evolution, still today give major contribution to the maintenance of the biosphere [1]. The unicellular photoheterotrophic transformable cyanobacterium *Synechocystis* sp. PCC6803, characterized by an intracellular thylakoid membrane, where both photosynthesis and respiration take place, is the first photosynthetic organism for which the complete genome sequence has been published [2].

*In vitro* or *in vivo* function is not known for any of the putative potassium channels identified in the genomes of over ten species of cyanobacteria [3], [4]. The only cyanobacterial ion channels characterized up to now are the prokaryotic glutamate receptor GluR0 [5] and the ligand-gated channel GLIC [6]. In general, the physiological role of bacterial channels is still largely unknown, except for bacterial chloride channel ClC [7], mechanosensitive channels [8] and *H. pylori* HpKchA, a putative potassium channel [9]. Potassium is the major intracellular cation in bacteria [10]. However, membrane potential adjustment rather than K^+^ uptake has been hypothesized to be the major function of K^+^ channels in prokaryotes, although direct proof is still missing [3]. In *Synechocystis* a Ktr-like system encoded by *slr1509*, rather than a *bona fide* channel, seems to be the main responsible for potassium uptake [4], [11].

In higher plant thylakoids several potassium-conducting cation channel activities have been described [12]–[15]. Furthermore, a putative potassium channel protein has been found in thylakoids of spinach [16]. Unfortunately, the molecular identity of the protein(s) responsible for these activities is unknown, as is the nature of the putative channel protein.

In the present study we characterized a novel cyanobacterial potassium channel. Furthermore, our work identifies its homolog in higher plants from molecular point of view and indicates its localization in the thylakoid membrane.

## Results

### Bioinformatic analysis of SynK putative potassium channel

**Figure 1:**
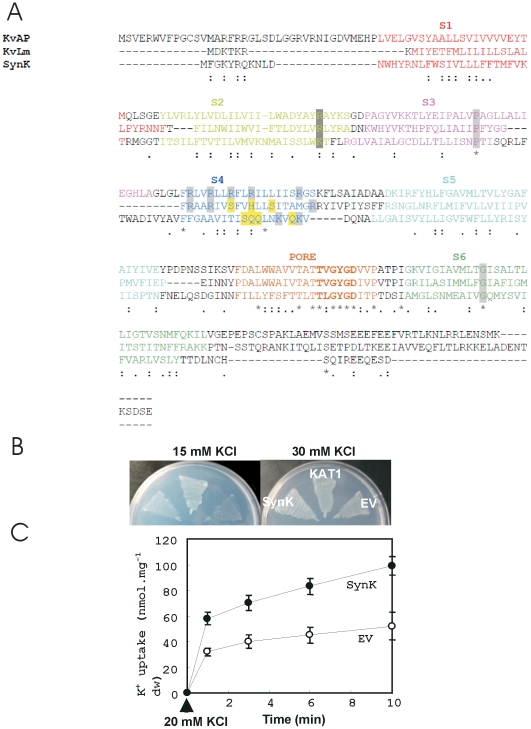
SynK protein permits potassium flux, as revelaed by its expression in K^+^-uptake deficient *E. coli* strain LB2003. **A**) SynK (*Synechocystis sp*. PCC 6803; gi:16331771) is characterized by selectivity filter sequence (bold characters) in pore region (brown characters) and by six predicted transmembrane segments (S1–S6 represented by different colours). ClustalW (1.83) alignments of SynK sequence with KvAP from *Aeropyrum pernix* (gi:14601099) and KvLm from *Listeria monocytogenes* (gi:16411529), two depolarization-activated prokaryotic potassium channels, is shown. “*” - identical residues in all aligned sequences; “:”–conserved and “.” - semi-conserved substitutions. Definition of S1–S6 segments in latter proteins is shown according to [19] and [20], in different colours. In SynK S1–S6 segments were defined according to secondary structure predictions (Porter, SPLIT4, TMHMM2 algorithms) and adjusted taking into account delimitation of α-helices as inferred from crystal structure of KvAP (according to [20]). Conserved residues, functional in Kv gating, are shaded grey. Please note the presence of some of the highly conserved residues in the sensor sequence of Kv channels, such as K63 in S2 and P86 in S3 in SynK. Polar residues (S and Q) in S4 are shaded yellow. **B**) Complementation growth test of *E. coli* LB2003 cells by *SynK*. *E. coli* LB2003 was transformed with plasmid harbouring pPAB404-*SynK* or empty vector. KAT1, an *Arabidopsis* K channel, was also included as a positive control. Transformants were grown on media supplemented with different concentration of KCl. **C**) Potassium uptake by K^+^-depleted *E.coli* containing *SynK* or empty vector. Net K^+^ uptake by *SynK*-expressing *E. coli* LB2003 cells and control cells harbouring empty vector were measured at 20 mM KCl. Data are averages ±SD of results from four independent experiments.

We identified in the genome of *Synechocystis* sp. PCC 6803, a hypothetical protein of unknown function (*slr 0498*) by homology search using the highly conserved selectivity filter [17], [18] amino acid sequence (T-X-G-[Y-F-L]-G-D) as a query sequence. SynK was predicted to harbour six membrane-spanning segments (S1–S6) and a pore region between helices S5 and S6 (Figure 1A). The aminoacid sequence of two other well-characterized prokaryotic 6 TM potassium channels, KvAP [19] and KvLm [20], is also shown for comparison. Although sequence homology between SynK, KvAP and KvLm is not high, some residues known to be important for channel gating are also conserved in SynK (Figure 1A). Positive charges present in the S4 helix of KvAP determine voltage-dependent gating [19]. KvLm has only two positive charges in S4, but shows strong voltage-dependence [20]. SynK does not display evenly spaced positive charges in the predicted S4 segment, nor does it contain regulatory domains. On the basis of bioinformatic analysis, SynK may be classified as a “core-only”, six-TM, putative potassium channel protein (see also ref.3). The closest homologues of SynK are found in other cyanobacteria species (Figure S1).

### SynK forms functional, potassium-conducting protein, when expressed in a K^+^-uptake-system deficient *E.coli* strain

An *E.coli* K^+^ uptake–deficient mutant has been successfully used to study potassium transport activity of transporter systems from plants [21] as well as from *Synechocystis* [22]. Here we cloned the *Synechocystis SynK* gene into the *E. coli* strain LB2003, carrying mutations in genes encoding the three major K^+^ uptake systems, Kdp, Trk, and Kup [23]. Thus, LB2003 does not grow at K^+^ concentrations ≤10 mM, due to negligible K^+^ uptake activity at potassium concentrations in the low millimolar range. Complementation test on solid media shows that *SynK*-expressing *E. coli* LB2003 cells grew well on a medium supplemented with 15 mM KCl, whereas *E. coli* cells harbouring empty vector did not (Figure 1B). Time course uptake experiment shows that K^+^ influx by *SynK*-expressing cells was higher compared to that of cells containing empty vector (Figure 1C). Net potassium uptake measurements by K^+^-depleted *E. coli* cells in the presence of 10 to 80 mM KCl revealed *V*_max_ values of 553 and 460 nmol min^−1^ g^−1^ dry weight for *SynK*-expressing cells and for the control cells, respectively (Figure S2). These data suggest that SynK may mediate K^+^ uptake when expressed in *E. coli*.

### Expression of SynK in CHO cells gives rise to potassium-conducting current

Additional functional characterization was performed in a mammalian cell system, given that SynK did not express in oocytes (Uozumi et al, unpublished). No electrophysiological studies have been performed on any cyanobacterial membrane until now. However, cloned prokaryotic channels have previously been shown to function in both heterologous expression systems e.g. [5], [6], [20], [24] and in artificial lipid bilayers e.g. [19], [25].

**Figure 2 pone-0010118-g002:**
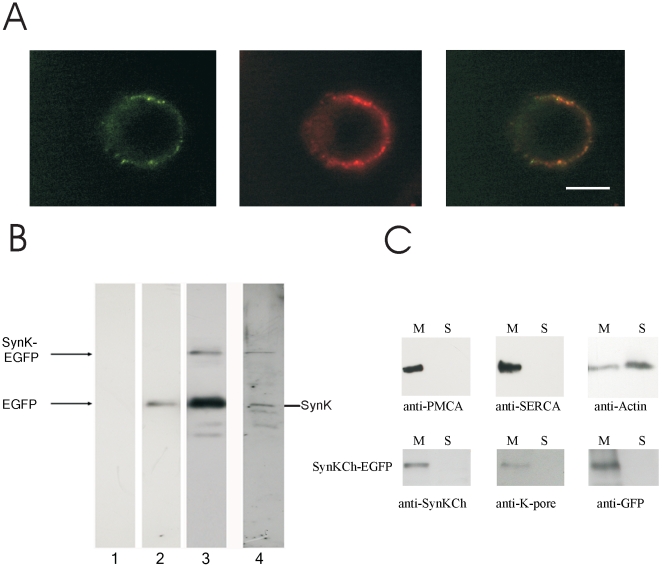
Expression of SynK in Chinese Hamster Ovary cells. **A**) SynK-EGFP fusion protein expression in CHO cell plasma membrane, revealed by fluorescence microscopy. Fusion protein (left image) and PM-specific Vybrant DiI dye (central image) co-located as indicated by overlapping image (right). Representative images are shown. Bars: 10 µm. Unequal distribution of Vybrant DiI may be due to preferential concentration of dye in rafts or to rapid vesicular uptake. **B**) SynK-EGFP is expressed with predicted molecular weight in CHO cells. Untransfected cells (lane 1) and CHO cells transfected with pEGFP-N1 (lane 2) or pSynK-EGFP (lanes 3, 4) were lysed 72 h after transfection, and 50 µg (lanes 1, 2, 4) or 100 µg (lanes 3) total proteins were loaded. Membranes were developed with anti-GFP (lanes 1–3) or anti-SynK (lane 4) primary antibodies. Arrows: positions of EGFP (28 kDa), SynK (27 kDa) and SynK-EGFP (54 kDa) proteins. **C**) SynK fusion protein is revealed in membraneous fraction. The purity of soluble and membrane fractions obtained from transfected CHO cells was checked by antibodies against marker proteins of the plasmamembrane (PMCA) (140 kDa), endoplasmatic reticulum (SERCA) (110 kDa) and cytosol (actin) (42 kDa) (upper panels). Actin is found also in the membraneous fraction because it is in part associated to organelles and cytoskeletron. SynK-EGFP fusion protein is present in the membraneous fraction (lower panels). Equal volumes of pellet and supernatant fractions, obtained as described in the Material and Method section, were loaded on SDS-PAGE (25 µl for samples developed with anti-SynK and anti-KPORE and 15 µl for those developed with anti-GFP antibody).

The sequence of SynK was isolated from the *Synechocystis* genome by PCR and a SynK-EGFP (enhanced green fluorescent protein at C-terminus) fusion protein was expressed in CHO (Chinese hamster ovary) cells. Mammalian HEK and CHO cells do not have significant endogenous potassium current, and are suitable for the expression of prokaryotic and even the viral channel Kcv e.g. [5], [26]. Green fluorescence of SynK-GFP was clearly associated with the plasma membrane (PM) (Figure 2A and Figure S3). Immunoblotting with anti-GFP antibody as well as by a specific anti-SynK antibody (Figure S4) revealed the presence of a product with the expected molecular weight of the fusion protein (for SynK and SynK-EGFP fusion proteins predicted MWs are 26445 and 53979 Da, respectively) (Figure 2B). However, lower MW products, corresponding to either EGFP alone (28 kDa), to SynK alone (27 kDa) or to degradation products of the fusion protein, were also observed and may account for the fluorescent signal observable in the cytosol of some cells (Figure S3 and not shown). Western blot of separated membrane and soluble fractions from transfected cells showed the presence of the 54 kDa fusion protein exclusively in the former one indicating that the correctly translated product is inserted into the membrane (Figure 2C). The same protein was also recognized by another antibody which was developed against the common selectivity filter sequence of potassium channels (anti-KPORE, Figure S5 for details), confirming that anti-SynK recognizes a potassium channel protein.

**Figure 3:**
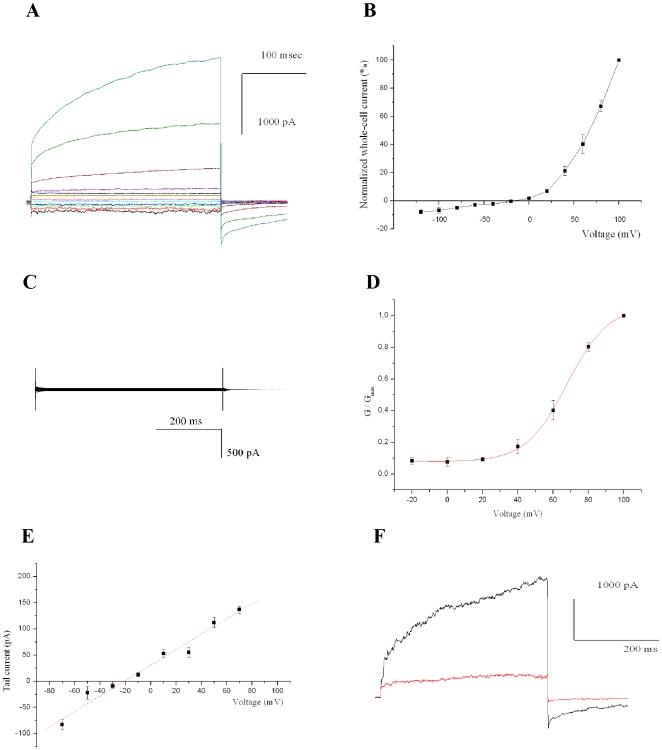
SynK functions as a potassium channel in CHO cells. **A**) Representative whole-cell currents in a pSynK-EGFP-transfected fluorescent cell, elicited by application of voltage steps of 300 ms duration, from −140 to + 100 mV in 20-mV steps, from a holding potential of −50 mV. Pulses were applied every 45 seconds, allowing complete deactivation of the channel. Different colours refer to different applied voltages. **B**) Current-voltage relationship. Peak currents normalized to current measured at +100 mV (n = 6, SEM values are reported). **C**) as in A), but from a control, pEGFP-N1-transfected cell. **D**) Boltzman fit of G/G_max_ (n = 6). **E**) Determination of selectivity from tail currents, elicited by stepping voltage for 400 ms to +60 mV, followed by application of −100 to + 100 mV in 20-mV voltage steps for 400 ms. Tail currents are reported as function of voltage. Reversal potential is −21±4 mV (n = 4). In A) to E) bath and pipette solutions contained 150 mM NaCl, 70 mM KCl and 134 mM KCl, respectively. **F**) Current recorded in K^+^-gluconate solution at +100 mV, before (black) and after (red) addition of 15 mM Cs^+^ to bath. Results are representative of 4 experiments.

Transfected CHO cells were identified by green fluorescence and analyzed by patch clamping in whole-cell configuration. SynK gave rise to an outwardly rectifying current (Figure 3A and B) (n = 32). Cells either left untransfected or transfected with control plasmids never displayed such a current (Figure 3C) (n = 40). The SynK current had an instantaneous and a slowly activating component (Figure 3A), the latter having an activation voltage of +67 mV as determined from the Boltzman fit of the G/G_max_ curve (Figure 3D). SynK activity was selective for cations as indicated by the fact that it was observed in the presence of potassium gluconate (Figure 3F, and not shown). Tail current analysis revealed a reversal potential (E_rev_) of −21±4 mV (n = 4) which is consistent with potassium selectivity (the predicted E_rev_ for a perfectly selective channel in our ionic conditions is −23 mV) (Figure 3E). Furthermore, SynK was blocked by 15 mM cesium (Figure 3F) and could not be observed with solutions containing tetraethylammonium chloride (n = 10, not shown), a general potassium channel blocker [17]. To further prove that the activity observed was due to SynK, we also transfected CHO cells with SynK bearing a single point mutation in the selectivity filter GYGD (in the mutant tyrosine 181 was changed to alanine). K^+^ channels with GAGD sequence are known to be expressed, but are unable to conduct a current e.g. [27]. The mutant SynK was efficiently expressed and targeted to PM in CHO cells (Figure S3) but did not give rise to current (n = 6) (not shown). These data indicate that SynK does form a potassium selective channel.

### SynK is located to both thylakoid and plasmamembrane in cyanobacteria

**Figure 4:**
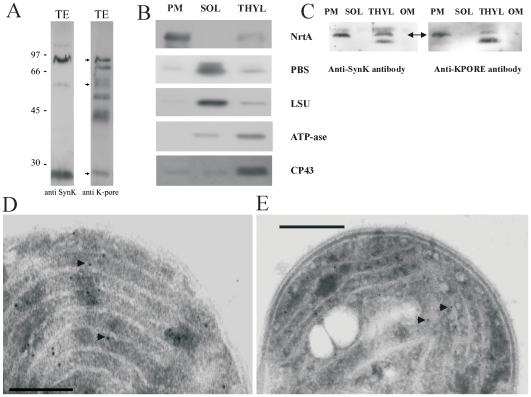
Localization of SynK in *Synechocystis*. **A**) Whole-cell cyanobacterial lysates containing 0.1 µg chlorophyll/lane were loaded on SDS-PAGE without urea and blotted with anti-SynK (1∶2500 dilution) (lane 1) and anti-KPORE (1∶10000) (lane 2) polyclonal antibodies. Apparent MWs of monomer, SDS-resistant dimer trimer and tetramer forms correspond to 26, 52, 76 and ca. 110 kDa. The anti-KPORE antibody, as expected, given the predicted presence of various potassium channels in this organism, recognized other proteins as well (lane 2). **B**) Plasmamembrane (PM), soluble (SOL) and thylakoid membrane (THYL) fractions were isolated from *Synechocystis*. The resulting fractions were checked for purity by using antibodies against markers of the plasmamembrane (NrtA), of the soluble fraction (PBS: allophycocyanin; LSU: large subunit of Rubisco) and of Thylakoid (ATP-ase and CP43). Cross-contamination to small extenct can be observed. 20 µg of proteins/lane. **C**) The obtained fractions were assayed for SynK content by using anti-SynK (left panel) and anti-KPORE (right panel) antibodies. 20 µg of proteins loaded/lane. The apparent MWs of the observed bands are 26 kDa (arrow) in the PM fraction and 26 and 24.5 kDa in the THYL fraction. **D**) Anti-SynK antibody used for immunogold electron microscopy confirms location of SynK protein in thylakoids (white membraneous structures). Arrows emphasize some of the gold particles. Bar: 200 nm. **E**) As control, anti-CP43 was used. Bar: 500 nm.

Determination of the subcellular localization of a protein is an important step toward understanding its function. To address this point, we obtained a polyclonal antibody against a recombinant protein expressed in *E.coli*, comprising the first 144 amino acids but not the pore region (Figure S4). The antibody recognized a band with the predicted molecular weight of 26 kDa (Figure 4A) with an efficiency comparable to that of the commercially available anti-ATP-ase antibody (Figure S6). Under certain solubilization conditions, known to permit visualization of SDS-resistant multimeric forms of prokaryotic potassium channels e.g. [28], bands with apparent molecular weights of 26, 52, 76 and 110 kDa were detected (Figure 4A). These values match the predicted masses for the monomeric (26445 Da) and multimeric forms of SynK, and point to a tetrameric organization. The use of anti-KPORE antibody further confirmed that anti-SynK recognized a potassium channel in cyanobacteria. To investigate the location of SynK protein, cytoplasmic and thylakoid membranes were isolated. Control blots performed with antibodies against marker proteins of the various fractions (Figure 4B) indicated that the cross-contamination in our preparation is low. At equal loaded protein quantity of plasmamembrane (PM), soluble (SOL), thylakoid (THYL) and outer membrane (OM) fractions, both anti-SynK and anti-KPORE antibodies recognized a 26 kDa band in the PM fraction as well as a 26 kDa band and a 24.5 kDa band in the thylakoid fraction (Figure 4C). These proteins are integral membrane proteins as they are resistant to alkaline extraction (not shown). Immunogold electron microscopy confirmed localization of the channel in the thylakoid and in the plasmamembrane (Figure 4D). As a positive control we used a specific antibody against CP43 protein of Photosystem II (Figure 4E), known to be located exclusively in the thylakoid membrane [29] and as negative control we used gold-coupled secondary IgG (Figure S7). Please note that the position of the anti-CP43-coupled gold particles with respect to the thylakoid membrane (white membraneous structure) is comparable to that obtained with anti-SynK antibody.

### A homolog of SynK is present in the thylakoid membrane of *Arabidopsis*

**Figure 5:**
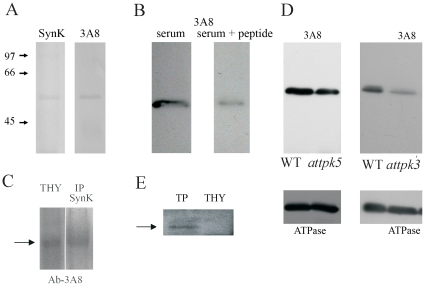
SynK homolog TPK3 is located in the thylakoid membrane of *Arabidopsis*. **A**) SynK and the monoclonal antibody 3A8 against TPK3/5 recognize the same, 54 kDa band in *Arabidopsis* wild-type thylakoids (proteins corresponding to 30 µg chorophyll were loaded). **B**) Intensity of the 54 kDa band decreased when the antibody was preincubated with 300 µM immunogenic peptide. The two lanes (30 µg Chl/lane) are from the same blot and were processed together. **C**) Thylakoids isolated from WT *Arabidopsis* plants were immunoprecipitated with anti-SynK antibody and blotted with 3A8 monoclonal antibody. **D**) Thylakoids (30 µg Chl/lane) isolated from wild type and TPK5-knock-out (left panel) and TPK3-knock-down (right panel) plants were loaded and assayed with the monoclonal antibody. The same membranes were stripped and reblotted with anti-ATP-ase to check for equal loading. **E**) Tonoplast and thylakoid fractions (20 µg of total protein of each) were loaded and developed with anti-TIP1.1 antibody (TIP1.1 is indicated by arrow at 28 kDa). In A, C and E nitrocellulose membranes and the BCIP/NBT (Sigma) development system, while in B and D PVDF membrane and ECL system was used.

The closest homolog of SynK in *Arabidopsis* is TPK3 (Score: 41,2; expect value: 3e-08, 36% identity, 51% positivities; Figure S8), which has a consensus prediction for localization in chloroplasts (http://aramemnon.botanik.uni-koeln.de/). TPK5 also shows some sequence similarity to SynK, and has a very strong predicted targeting for chloroplast according to several algorithms. Although electrophysiological and biochemical evidence suggest the presence of potassium-conducting channel(s) in higher plant thylakoid membrane, the molecular nature of this(ese) protein(s) is unknown. Given that the SynK antibody was developed against the first 144 amino acids of the protein, i.e. a region comprising stretches of amino acid sequences which are conserved also in TPK5 and TPK3, we predicted that *a priori*, the anti-SynK antibody might recognize both proteins in *Arabidopsis* thylakoids, if these proteins were located in that membrane system. Anti-SynK antibody revealed a protein with an apparent MW of 54 kDa in thylakoids isolated from *Arabidopsis* (Figure 5A). Membrane proteins often display a migration resulting in different MW from that predicted. Since an MW of 54 kDa is somewhat higher than that predicted for TPK5 and TPK3 (46,3 and 48,7 kDa, respectively), we developed a monoclonal antibody (3A8) against a region conserved in *Arabidopsis* TPK3/5 but not in other members of the TPK family. 3A8 gave visible reaction already with 100 ng of the immunogenic peptide in dot blot (not shown). The 54 kDa band was recognized by both anti-SynK and 3A8 (Figure 5A) and also by other two monoclonal antibodies developed against the same peptide and by anti-KPORE (not shown). The specificity of the recognition by 3A8 is indicated by the significant decrease of the intensity of the band when the antibody was pre-incubated with its immunogenic peptide prior to blot development (Figure 5B). The identified protein is an integral membrane protein (Figure S9). Furthermore, the 54 kDa protein, pulled down by anti-SynK antibody from *Arabidopsis* thylakoid, was recognized by the monoclonal anti-TPK3/5 antibody (Figure 5C). To further prove the nature of the 54 kDa band, we performed Western blots on thylakoids isolated from TPK5-knock-out *Arabidopsis* mutant (Figure 5D). The intensity of the 54 kDa band was not significantly altered in the thylakoid membrane isolated from the knock-out plant with respect to that observed in WT thylakoids. Given that in the TPK5-knock-out plants transcripts of TPK5 were absent (not shown), the 54 kDa band in the mutant plant was attributed to TPK3. Therefore we checked for the presence of this band in plants with a t-DNA insertion in the TPK3-encoding gene. t-DNA insertion mutants are only available in the UTR or in the promoter regions for TPK3. UTR (untranslated regions) may affect efficiency of translation and the lifetime of transcripts. The transcript level of TPK3 was slightly reduced in the UTR-insertion mutant with respect to that found in wild-type (not shown). In thylakoids isolated from these plants there was a decrease of the intensity of the 54 kDa band, but complete disappearance could not be observed, being compatible with the presence of a reduced amount of TPK3. Given that most TPK channels, including TPK1, have been proposed to be located in the membrane around the vacuole, i.e. in tonoplast in plant cells [30], we checked for contamination of our thylakoid preparation by tonoplast. In Figure 5E the anti-TIP1.1 antibody raised against an aquaporin located to tonoplast [31], recognized a 28 kDa band in isolated tonoplasts, but not in thylakoids. As a further control, the localization of TPK1 in *Arabidopsis* cells was assayed by using a specific anti-TPK1 monoclonal antibody. Western blot analysis of vacuolar and thylakoid fractions revealed the presence of a 51 kDa band only in vacuoles isolated from WT but not in those obtained from TPK1 knock-out plants, confirming tonoplast location of TPK1 and indicating that TPK proteins might migrate with a higher than predicted MW (Figure S10).

## Discussion

In the present work we report cloning and functional characterization of a novel potassium channel of cyanobacteria. The SynK protein, identified as putative potassium channel by bioinformatics, was shown to mediate potassium transport when expressed in *E.coli* LB2003 and gave rise to potassium-selective current when studied in Chinese Hamster Ovary cells. Specific anti-SynK antibody localized the channel protein both in thylakoid and in plasmamembrane in *Synechocystis* cyanobacteria. SynK is thus the first potassium channel identified in the thylakoid membrane from molecular point of view. Furthermore, SynK seems to be the ancestor of a TPK family member in *Arabidopsis*, which we show to be located in thylakoids of higher plants.

SynK is shown here to function as potassium-conducting channel when expressed in heterologous systems (Figures 1–3), although structural determinants of voltage sensitivity in SynK and factors determining the instantaneous component remain to be clarified. Data of Figure 4 indicate SynK to be located in both plasma and thylakoid membranes in *Synechocystis*. Recently, we have identified another ion-conducting pathway, a sodium/proton antiporter, in the thylakoid membrane of the same organism [32]. Dual localization of several proteins and ion channels have been described in eukaryotic systems e.g. [33]–[35]. The targeting mechanisms are not well known in cyanobacteria, but according to one model, proteins may be initially targeted to either membrane and sorted afterwards, possibly by vesicle transport [29]. Recently, the Tat protein transport system was described to function in both membrane systems [36]. In the thylakoid membrane fraction the anti-SynK antibody detected two bands, one with a slightly lower MW than that predicted (Figure 4C). Whether this lower MW band corresponds to a mature form of the thylakoid-targeted protein or to a partially degraded protein remains to be determined.

Chloroplasts are descendents of an ancestral endosymbiont of cyanobacterial origin e.g. [37], [38]. Nuclear genes coding for chloroplast proteins involved in photosynthesis and organelle biogenesis have been identified. A recent work identified other nuclear-encoded chloroplast proteins of endosymbiont origin by using functional orthogenomics [35]. Our data suggest that SynK may be an ancestor of TPK3 which is a member of the two-pore potassium channel family in *Arabidopsis* [39]. When BLAST analysis is performed, TPK3 is the closest homolog of SynK in the whole *Arabidopsis* genome and *vice versa*, according to Aramemnon. The evolutionary origin of eukaryotic tandem-pore channels is still elusive but according to one hypothesis, 6TM prokaryotic PNBD-less potassium channels (like SynK) might have given origin to TPK channels [40]. A conserved pore region feature (presence of YF residues) in both SynK and plant TPK channels further point to an evolutionary link between the two proteins (Figure S11).

Our findings indicate the presence of TPK3 protein in the thylakoid membrane (Figure 5). Independently of whether SynK is the precursor of TPK3 or not, this is the first thylakoid-located cation channel identified from molecular point of view in higher plants (in addition to proton-conducting F_0_/F_1_ ATP-ase). Given that the electrophysiological activity of TPK3 has not been described up to now, it is difficult to predict which of the previously described electrophysiological activities [12]–[15] can be assigned to TPK3 protein. In any case, the thylakoid localization of this protein opens the way to functional characterization of this still putative channel. Despite a consensus prediction for chloroplast localization of TPK1, TPK2 TPK5 and TPK3 (see Aramemnon site), these proteins have previously been shown to be targeted to the vacuolar membrane of protoplasts from *Arabidopsis* cultured cells that transiently expressed AtTPK in fusion with GFP or YFP under the control of the cauliflower mosaic virus (CaMV) 35S promoter [30]. Interestingly, AtTPK3 fusion protein accumulated also in additional, non-identified internal membranes when using this system (Figure 2b of ref. 30). We would like to point out that we detect AtTPK3, shown to exhibit high transcript level [30], in thylakoids obtained from genetically non-manipulated *Arabidopsis* plants, by using a specific monoclonal antibody. Thus, observation of the protein in thylakoids due to possible overexpression-induced mistargeting can be excluded. Our results do not exclude localization of TPK3 in other membranes as well, nor they exclude the presence of other channels as well in thylakoids. SynK and TPK3 might be involved counterbalancing cation fluxes from the lumen towards the stroma during photosynthesis, which would permit dissipation of the transmembrane potential but not that of the pH gradient [12], [15], [41]. Presuming the same orientation of SynK in the CHO plasma membrane and in thylakoids, at positive voltages of the thylakoid (proposed to reach +70 mV on the lumenal side during proton flux into the lumen [42]) SynK could permit the quick exit of potassium from the lumen. Direct genetic proof in favour of the “counterbalance” hypothesis is still missing, due also to the fact that cation channels have not been identified from a molecular point of view neither in cyanobacterial thylakoid nor in that of higher plants.

In summary, we report the molecular identification of two thylakoid-located potassium channels, SynK in cyanobacteria and TPK3 in *Arabidopsis*. SynK represents the first cyanobacterial core-only type potassium channel, and seems to be the anchestor of TPK3 of the two-pore potassium channel family. Our results open the way for understanding the physiological roles of these thylakoid channels and for determining their role, if any, in the regulation of photosynthesis.

## Materials and Methods

Strains and growth conditions are described in supplementary Text S1. Expression of SynK in *E.coli* and measurement of K^+^ uptake was performed according to [21] and [43]. Expression of SynK in CHO cells was performed according to [44]. DNA constructs and transformation of *Synechocystis* sp. PCC 6803 as well as plant growth, genotyping and transcript analysis of *Arabidopsis* are detailed in the suplementary material. Thylakoids from plants were isolated as described [45]. Membrane fractionations of CHO cells, cyanobacteria and *Arabidopsis* were performed according to [46], [47] and [48], respectively. Immunoprecipitation, electron microscopy and immunogold labelling were performed according to [49] and [50], respectively. Patch clamp analysis is according to [34], [44] and is detailed in supplementary Text S1.

## Supporting Information

Text S1 (0.04 MB DOC)

Figure S1 Closest homologues of SynK are found in cyanobacteria. A) The closest homologues of SynK (Syn, *Synechocystis* sp. PCC 6803; gi:16331771) are found in other cyanobacteria species. Sequence alignment (ClustalW (1.83) algorithm) of SynK, of a hypothetical protein (Lyng, *Lyngbya* sp. PCC 8106; gi:119457762) and K^+^ channel pore region (Croco, *Crocosphaera watsonii* WH 8501; gi:46119130). “*” - identical residues in all aligned sequences; “:” - conserved and “.” - semi-conserved substitutions. BLAST analysis revealed E values (number of hits expected to be found by chance) of 2×10–24 and 4×10–19 and positivity over length of aligned sequence of 55% (223 amino acids) and 56% (207) when compared SynK with *Lyngbya* and *Crocosphaera watsonii* proteins, respectively. Typical selectivity filter for potassium is in green. Glycine in S6, important for gating is in yellow.
(0.02 MB DOC)

Figure S2 Potassium uptake by K^+^-depleted *E.coli* containing SynK or empty vector. Net potassium uptake measurements by K^+^-depleted *E. coli* cells in the presence of 10 to 80 mM KCl revealed Vmax values of 553 and 460 nmol min−1 g−1 dry weight for SynK-expressing cells and for the control cells, respectively Lineweaver-Burk plot of K^+^ uptake data obtained from four independent experiments is shown.
(0.02 MB PDF)

Figure S3 Expression of SynK and SynK mutant in Chinese Hamster Ovary cells. SynK-EGFP WT and mutant (non-conducting mutant with GAGD instead of GYGD in the pore region) fusion protein expression in CHO cell plasma membrane was revealed by confocal microscopy. Images with GFP fusion proteins (left images) and FM4-64 dye (central images) and merged signals (right images) are shown for WT SynK-GFP (upper panels) and mutant SynK-GFP (lower panels). Graphics shown beside the merged images represent profile plots of GFP (green) and FM4-64 (red) fluorescence intensity as a function of the distance for a particular region of interest (ROI), from inside the cell (in) to outside (out). Peaks falling in the same region correspond to co-localization.
(0.48 MB PDF)

Figure S4 Anti-SynK antibody recognizes recombinant and native SynK. Recombinant protein (144 N-terminal amino acids of SynK fused with a 6 His-tag at C-terminus) was expressed in *E. coli* and purified as described in Materials and Methods. Protein was purified as a 30-kDa dimer (see lane 2). 30-kDa protein, recognized by anti-His antibody (not shown), was used for antibody production. Pre-immune antiserum did not recognize either purified 30 kDa protein (lane 3) or proteins in cyanobacteria whole-cell lysate (lane 4); serum from immunized rabbit clearly reacted with the recombinant protein (lane 5) and recognized SynK of 26 kDa in whole-cell lysate (in cells containing 0.1 µg chlorophyll) even at 1∶5000 dilution (lane 6).
(0.16 MB DOC)

Figure S5 Anti-KPORE antibody recognizes other potassium channels. Anti-KPORE antibody was used at 1∶10000 dilution on whole-cell lysate of Jurkat lymphocytes, known to express Kv1.3 channel with apparent MW of 65 kDa (Magic Marks loaded on lane 1). Same bands were recognized by anti-KPORE (lane 2) and by a specific antibody against Kv1.3 (1∶200) (lane 3) in SDS-PAGE with 6 M urea. 50 µg total proteins were loaded. Anti-KPORE antibody also recognized purified GST-Kv1.3 protein (lane 4, 10 µg loaded, predicted MW 87 kDa) (production of GST-Kv1.3 is described in Gulbins et al, Biochim. Biophys. Acta, in press). Anti-KPORE antibody also recognized KCa3.1 in HCT116 colon cancer cell line (not shown), and monomeric as well as multimeric forms of the purified Kcv viral potassium channel (not shown) and of purified KvAP (kindly provided by P.Facci, not shown).
(0.07 MB DOC)

Figure S6 Anti-SynK antibody efficiently recognizes SynK in whole-cell lysate of cyanobacteria. Cells corresponding to the O.D. (at 730 nm) shown on the figure were solubilized in SB and loaded on SDS-PAGE. The blot was first developed with anti-SynK antibody and after re-stripping with anti-ATP-ase antibody (Agrisera). Efficiency of anti-Synk and anti-ATP-ase antibodies is comparable.
(3.76 MB PDF)

Figure S7 Secondary antibody does not label cyanobacteria in immunogold electron microscopy. As control, only secondary IgG was used. Bar: 500 nm.
(0.05 MB PDF)

Figure S8 Sequence homology between cyanobacterial SynK and *Arabidopsis* TPK3 (At4g18160). Aminoacid sequence alignments obtained by T-COFFEE algorithm. “*” - identical residues in all aligned sequences; “:” - conserved, “.” - semi-conserved substitutions.
(0.21 MB PDF)

Figure S9 The 54 kDa protein is an integral membrane protein. Thylakoids (100 mg total proteins) were subjected to alkaline extraction (0.2 M Na2CO3 for 30 minutes), pelleted and both pellet and supernatants were loaded. The 54 kDa band is not present in the supernatant fraction indicating that it is an integral membrane protein. Blots were developed with the indicated antibodies.
(0.10 MB PDF)

Figure S10 TPK1 locates to tonoplast in *Arabidopsis*. A specific monclonal antibody was used to reveal location of TPK1 in WT and atkco1 plants. Cells were fractionated and loaded on continuous sucrose gradient. Fractions positive for tonoplast TIP1 (VAC) or for thylakoid membrane D2 (THYL) were loaded. TPK1 is visible only in the vacuolar fraction of WT cells (at 50 kDa). An aspecific recognition is seen at approx. 35 kDa in thylakoids in both WT and mutant organisms.
(0.06 MB PDF)

Figure S11 Pore region and YF residues are highly conserved between SynK and TPK channels of *Arabidopsis*. Voltage-gated Kv and KCNQ channels are characterized by a conserved pore region feature, namely, the presence of two tryptophans in tandem (W67 and W68 in KcsA) (Minor DL (2001) Potassium channels: life in the post-structural world. Current Opinion in Structural Biology, 11: 408–414). In plant shaker-like inward rectifier channels, the second tryptophan is highly conserved and the first is replaced by a tyrosine. These same positions are strongly conserved within other families of potassium channels, however, as different residues. Animal Kir channels harbour LF or SF residues in the same position (Minor 2001). Instead, in animal two-pore channels, in viral Kcv as well as in all plant two-pore channels the same positions are occupied by tyrosine and phenylalanine (YF). SynK has the same YF aminoacids in the corresponding position, further suggesting that SynK might have given origin to two-pore channels during evolution. Interestingly, GORK and SKOR outwardly rectifying voltage-dependent channels, also harbour YF residues in the corresponding position but, in contrast to TPK3, do not show significant homology with SynK. Aminoacid sequence alignments obtained by T-COFFEE algorithm. “*” - identical residues in all aligned sequences; “:” - conserved, “.” - semi-conserved substitutions. YF residues, typical of Kcv, animal and plant two-pore potassium channels are indicated. At4g01840: TPK5; Atg1g02510: TPK4; At4g18160: TPK3; At5g46370: TPK2; At5g55630: TPK1.
(0.03 MB PDF)
