# Supplementary material for: A Novel Potassium Channel in Photosynthetic Cyanobacteria
Source: PLoS One. 2010 Apr 12;5(4):e10118. doi: 10.1371/journal.pone.0010118 (PMC2853561; doi:10.1371/journal.pone.0010118)
Supplement: Text S1 — (0.04 MB DOC) [file pone.0010118.s001.doc]

# Zanetti et al, Supplementary Material

**Materials and Methods**

*Strains and growth conditions*

The wild-type *Synechocystis* sp. glucose-tolerant strain PCC 6803 was grown at 30°C under white light (30 μmol of photons / m^2^sec) and shaking in standard BG-11 medium, buffered at pH 8.0 with 10 mM HEPES (*N*-2-hydroxyethylpiperazine-*N’*-2-ethanesulfonic acid) and supplemented with 10 mM glucose for photoheteroptrophic growth. For cultures grown on plates, the BG11 medium was supplemented with 1.5% agar and 0.3% sodium thiosulfate.

*Expression of SynK in E. coli defective in K^+^ uptake system*

Heat shock method was used for the transformation of *E. coli* strain LB2003 competent cells by pPAB404-*SynK*. For the complementation growth test, the cells of *E. coli* LB2003 containing the plasmids were plated on a synthetic solid medium containing 34 mM Na_2_HPO_4_, 17 mM NaH_2_PO_4_, 8 mM (NH_4_)_2_SO_4_, 0.4 mM MgSO_4_, 0.6 μM FeSO_4_, and 2% glucose , in the presence of 0.25 mM IPTG, 40 µg/ml of ampicillin, and various concentration of KCl. The plates were then subjected to an overnight incubation at 30°C. K^+^ uptake assay was essentially measured as described elsewhere [43] . *E. coli* LB2003 cells were cultured in a synthetic medium at 30°C. The cells were collected by centrifugation, resuspended in 120 mM Tris-HCl (pH 8.0) and 1 mM EDTA was added after the cell concentration was adjusted to an OD_578_ of 30. Subsequently, the cell suspension was shaken for 10 min at 37 °C, collected by centrifugation, and washed twice with 200 mM HEPES-NaOH (pH 7.5) and then resuspended in the same buffer. After shaking for 20 min at room temperature, the concentration of cells was adjusted to an OD_578_ of 3 with the same buffer. Ten minutes prior to the start of the K^+^ uptake measurement, 10 mM glucose was added to the suspension. One ml of the cell suspension was taken at an indicated time and transferred into a tube containing 150 µl of silicon oil, and subsequently centrifugated at 12,000 rpm for 1 min. The potassium content of cell pellet was determined by flame photometry.

*DNA Constructs and Transformation of Synechocystis sp. PCC 6803.*

The forward K+E (5’-gagccag**gaattc**atgtttggcaaatatcgac-3’) and reverse K+F (5'-ATGGGCTC**GGATCC**GATTCCTGTTCTTCC-3') primers, introducing restriction sites *EcoRI* and *BamHI* respectively, were used to generate a 725-bp PCR product including exactly the *slr 0498* open reading frame (ORF). The *EcoRI*-*BamHI* fragment, subsequently obtained by restriction digestion, was then cloned into the same sites of plasmid pEGFP-N1 (Clontech) to give plasmid pEGFP-SynK. In the new plasmid, the entire insert was completely sequenced to verify that no undesired mutation had been inserted by Taq polymerase (Platinum® *Taq* DNA Polymerase High Fidelity, Invitrogen). For production of the SynK-EGFP fusion bearing mutation Y181A, two overlapping fragments were generated in separate PCR amplifications, corresponding to the 5’ region (primers K+E and Y2Arev, 5'-TATGTCACCGGCGCCCAGGGTGG-3') and 3’ region (primers Y2Afor, 5'-ACCCTGGGCGCCGGTGACATAAC-3', and K+F) of the *slr0498* ORF. These two fragments were purified from oligonucteotides, mixed and used as template in a third amplification with primers K+E and K+F. The resulting band was then processed as described above.

The forward NtK-Nco (5’-TTCGAAT**CCATGG**TTGGCAAATATCGACAG-3’) and reverse Ntk-Xho (5’-ATAGGATATCCG**CTCGAG**TAAAAACCAAAAGAC-3’) primers, introducing restriction sites *NcoI* and *XhoI* respectively, were used to generate a 432-bp fragment by PCR amplification of plasmid pEGFP-SynK and subsequent restriction digestion. Plasmid pET-NtK5 was constructed by cloning this fragment into vector pET28b (Novagen), cut by the same enzymes; the cloned insert was controlled by sequencing.

*Plant growth, genotyping and transcript analysis*

*A. thaliana* wt (Columbia-0) and mutant plants were grown under short day (10/14 hours light/dark) with 100 μmol photons m^-2^ s^-1^ light at 23/18°C in a controlled growth chamber. Mutant plants are T-DNA insertion lines from SALK collection: *attpk5* (SALK_123690C), *attpk3* (SALK_090886) and *attpk1* (SALK_131790C). Mutants were genotyped by PCR. After genomic DNA extraction by using standard protocol, PCR primers were used as follow: *attpk5*-specific, 5’-TCGCTGTTGTTTTCGTCTTG-3’ and 5’-CAAAGGATCCCCCAAAAGAT-3’; *attpk3*-specific, 5’-CTCTTGGAAGGTGGCAGTGG-3’ and 5’-GTTGGGGCAGGTTTGTTGTT-3’; *attpk1*-specific, 5’-CGTCATGCTGGATATTTTGG-3’ and 5’-AGACGAAGGCACAAGCAAGT-3’; T-DNA-specific, 5’-CGATGGCCCACTACGTGAACCA-3’ and 5’-TGGTTCACGTAGTGGGCCATCG-3’. Total RNA was extracted from 100 mg of powdered leaves using the TriZol reagent (Gibco, Germany). After treatment with RNase-free DNase I (Ambion Ltd, UK), first strand cDNA was synthesized starting from 5μg of total RNA using the PowerScript™ Reverse Transcriptase (Clontech, USA). The presence of the transcripts was assayed by PCR using primers located in the exons located before and after the intron.

*Thylakoid membrane purification*

Thylakoids were isolated as described [45]. Briefly, *Arabidopsis* leaves were homogenized in 0.33 M sorbitol, 50 mM Tricine (pH 7.8), 5 mM MgCl_2_, and 10 mM NaCl. After filtering, the homogenate was centrifuged at 4,500 *g* for 2 min, and the pellet was resuspended in 50 mMTricine (pH 7.8), 5 mM MgCl_2_, and 10 mM NaCl. After centrifugation at 4,500 *g* for 10 min, the resulting thylakoids were resuspended in 100 mM sorbitol, 50 mM Tricine (pH 7.8), 5 mM MgCl_2_, and 10 mM NaCl.

*Purification of His-tagged SynK antigen and production of antisera*

The recombinant NtSynK-His protein (144 N-terminal amino acids of SynK fused with a 6 His-tag at the C-terminus) was expressed in *E. coli* strain BL21(DE3), transformed with plasmid pET-NtK5, by the addition of 0.7 mM IPTG (≈200 µg/liter of 2-h culture). The protein was purified from solubilized inclusion bodies by affinity purification on nitrilotriacetic acid resin (Qiagen) and subsequent electroelution of the corresponding band from SDS/12-17% PAGE with 6 M urea. The purified protein was used as antigen to immunize two rabbits by subcutaneous injections, with poly(A)-poly(U) as adjuvant and following standard immunization routes.

*Cell culture, fluorescence and confocal microscopy.*

Plasmid pEGFP-SynK was used to transfect CHO-K1 cells [44]. These were cultured at 37°C in a humidified atmosphere containing 5% CO_2_ on glass coverslips in Dulbecco’s essential medium (D-MEM) (GIBCO) containing 10% fetal bovine serum, 2 mM L-glutamine, 100 μg/ml streptomycin and 100 units/ml penicillin. 40%-confluent culture was transfected using Lipofectamine 2000 Reagent (Invitrogen). Following transfection, cells were cultured for 72 h at 37°C or at 30°C, in the presence of 1 mM Cs^+^, in order to prevent K^+^ ion imbalances. 1 mM Cs^+^ was not toxic for these cells. GFP fluorescence was examined with a Leica DMR microscope. The fluorescence filters were set at excitation 480 nm and emission 510 nm.

CHO cells were labeled with Vybrant DiI cell-labeling solution (Molecular Probes). This highly lipophilic dye can be analyzed by fluorescence microscopy: excitation 550 nm and emission 565 nm. For fluorescence microscopy, cells were assayed 72 hours after transfection with 3,2 μg plasmid DNA. Incubation with 0.125 μl of Vybrant DiI of cells in a small Petri dish (1 ml of culturing medium) for 8 minutes was followed by washing the cells three times.

For confocal images, obtained by using Leica LCS-SP2 system (Leica Microsystems, Heidelberg, Germany), FM4-64 (Invitrogen) was used as plasmamembrane dye according to product instruction. Co-localization with GFP signal was analyzed by Leica LCS software (Profile Plot). Cells were assayed 48 hours following transfection.

*Cell lysis, membrane fractionation, SDS-PAGE and immunoblot analysis.*

CHO cells, transfected with plasmids pEGFP-N1 (1.5 μg plasmid for every 2 million cells) and pEGFP-SynK (3.2 μg plasmid per 2 million cells) were lysed with SDS-PAGE loading buffer (LB) (50 μl LB for 10^6^ cells) in the presence of protease inhibitors. Total protein concentration was determined by the Bradford assay. For fractionation, transfected CHO cells (8 x 10^6^ cells) were washed with PBS, then harvested in 10 mM Tris/HCl pH 8, 2 mM EDTA, 2 mM PMSF, 1 mM DTT. Cells were disrupted by three cycles of freeze and thaw at -200°C (liquid nitrogen)/37°C. The membrane fraction was sedimented at 11.000 g for 30 min at 4°C. Pellet (membrane fraction) was resuspended in 100 µL sample buffer. The supernatant (soluble fraction) was precipitated with four volumes of ice cold acetone: after centrifugation for 5 min at maximum speed, the corresponding pellet was resuspended in 100 µL of SB [46]. Proteins were resolved by SDS-PAGE and electroblotted to nitrocellulose or PVDF (polyvinyldifluoridene) membranes. The blots were probed with primary antibody raised against GFP (1:2500), SynK (1:1000), KPORE (1:5000), PMCA (1:1000), SERCA (1:2500) and actin (1:5000). Primary antibody was detected with the ECL detection system (Pierce).

For cyanobacteria, cells were directly lysed in LB and loaded on SDS-PAGE containing or lacking 6 M urea, as specified. Samples were either boiled or not, in order to visualize multimeric or monomeric forms. Quantification was made by measuring OD_730_ and chlorophyll concentrations of the cultures. Proteins were blotted onto PVDF and membranes were decorated with primary antibodies in the presence of 1 % milk. Membrane fractionation of *Synechocystis* was performed by slight modification of the procedure described in Bolter et al, 1998 [47] . The blots were probed with anti-SynK (1:1000), anti-NrtA (kind gift of Prof. Pakrasi) (1:5000), anti-Rubisco large subunit (1:10000), anti-CP43 (1:1000), anti-ATP-ase (Agrisera) (1:10000) and anti-allophycocyanin (kind gift of Prof. Barbato) (1:1000).

For *Arabidopsis* cell membrane fractionation was performed according to [48]. Immunoprecipitation was performed as described in [49].

Anti-K-PORE antibody was produced against the synthetic peptide TTMTTLGYGD (Sigma). 3A8 monoclonal antibody was raised against the synthetic peptide IYSFNRDHYSGIET (Monoclonal Antibody Core Facility, EMBL). Anti-TIP1,1 (1:500) was a kind gift of Professor Raikhel. Anti-AtTPK1 antibody was raised against synthetic peptide DDVKIDEPPPHPSK (EMBL).

*Patch clamp analysis.*

Patch clamping experiments were performed in whole-cell patch configuration on control pEGFP-N1-transfected or pEGFP-SynK-transfected CHO cells [44]. Bath solution:150 mM NaCl, 70 mM KCl, 1 mM MgCl_2_, 2 mM CaCl_2_, 10 mM Hepes, pH 7.4 adjusted with NaOH. Pipette solution: 134 mM KCl, 2 mM MgCl_2_, 10 mM CaCl_2_, 10 mM K^+^/EGTA, 10 mM Hepes, pH 7.35 adjusted with KOH, unless specified otherwise. In experiments with TEA^+^, Na^+^ and K^+^ were replaced by TEA^+^. In experiments with gluconate, bath: 180 mM Kgluconate, 5 mM KCl, 1 mM MgCl_2_, 2 mM CaCl_2_, 10 mM Hepes, pH 7.4; pipette: 114 mM Kgluconate, 2 mM MgCl_2_, 10 mM CaCl_2_, 10 mM EGTA, 10 mM Hepes, pH 7.35. Potentials were applied and currents were monitored by an EPC-7 amplifier. Pulse protocol was applied and data analysis was performed by the PCLAMP8 program set. For tail current determination leak current was subtracted. Pipette resistance was 2-5 megaOhm. Data were low pass-filtered with an eight-pole Bessel filter with a cut-off frequency of 1 kHz. Intracellular voltages are reported and outward currents are plotted upwards.

*Electron microscopy and Immunogold labelling*

Pellets of cyanobacteria were fixed overnight at 4°C in 3% glutaraldehyde in 0.1 M sodium cacodylate buffer (pH 6.9) and then processed for electron microscopy according to [50]. Ultrathin sections, cut with an ultramicrotome (Ultracut, Reichert-Jung), were post-stained with lead citrate and examined under a transmission electron microscope (TEM 300, Hitachi) operating at 75 kV. For immunogold labeling, *Synechocystis* cells were fixed for 2 h in 4% paraformaldehyde and 0.25% glutaraldehyde in 0.1 M phosphate buffer (pH 7.2), post-fixed for 1 h in 1% osmium tetroxide in the same buffer, dehydrated in ethanol, and embedded in London resin white. Ultrathin sections picked up on gold grids were deosmicated with sodium metaperiodate, washed with 0.01 M PBS (pH 7.2), incubated for 20 min on 1% BSA in PBS, and treated with rabbit primary polyclonal antibody against SynK. After washing with PBS, sections were incubated with colloidal gold (15 nm) conjugated with goat anti-rabbit IgG. Sections were then stained with uranyl acetate followed by lead citrate, and examined under the electron microscope. A control experiment was performed by eliminating the incubation of sections with the primary antibody (Figure S7).
