# Supplementary material for: A Novel Potassium Channel in Photosynthetic Cyanobacteria
Source: PLoS One. 2010 Apr 12;5(4):e10118. doi: 10.1371/journal.pone.0010118 (PMC2853561; doi:10.1371/journal.pone.0010118)
Supplement: Figure S1 — Closest homologues of SynK are found in cyanobacteria. A) The closest homologues of SynK (Syn, Synechocystis sp. PCC 6803; gi:16331771) are found in other cyanobacteria species. Sequence alignment (ClustalW (1.83) algorithm) of SynK, of a hypothetical protein (Lyng, Lyngbya sp. PCC 8106; gi:119457762) and K+ channel pore region (Croco, Crocosphaera watsonii WH 8501; gi:46119130). “*” - identical residues in all aligned sequences; “:” - conserved and “.” - semi-conserved substitutions. BLAST analysis revealed E values (number of hits expected to be found by chance) of 2×10^–24^ and 4×10^–19^ and positivity over length of aligned sequence of 55% (223 amino acids) and 56% (207) when compared SynK with Lyngbya and Crocosphaera watsonii proteins, respectively. Typical selectivity filter for potassium is in green. Glycine in S6, important for gating is in yellow. (0.02 MB DOC) [file pone.0010118.s002.doc]

**Figure S1**

*Photo* ----------------------------MLLVSTSLVEVLPNKFGLLEYILEGFTALTFL *V.parahae* MRRTMVIGKKLKNINEENNFFYLTLALVILLLGSAMAQVVG--EGTIEHILQAFTVLTFI *V.vulnif.*  ----MKLDKALSNISEKNNFIYLTLALIMLLIAAALVQLLQ--DNVIEYVIQGIMVVIFI *Croco*  ------MNTTSRIYSPTNGYKYLFIDLIVLFLLIPFASIHR----SLSLIVSFCFLVTLL

*Syn*  -----MFGKYRQKNLDNWHYRNLFWSIVLLLFFTMFVKTRMG--GTITSILFTVTILVMV

:*:. :.. : :: : ::

VCLVSLRFDK---NWYRFMMTLAGCWLVATIIRNWLGIQQMDLIMLGLMFGFFFGTFKSV

VCFASLRFDK---TWSRFLYTLFGVWVLVIVIKTVFNIREMNVVMLALTFAFFFGTFKSI

VCFVSLRFDR---KWTHFLRGLAVVWIAAIAAKHLFHIKEMSILMLSLTFVFFYGTFQSL

LGLNTLAFPKRVLFLFRFFATLGFISDIIIFPNSQYLTDLSSLMSYSFYGIFYILVILAI

KNMAISSLWKT--FLRGLVAIALGCDLLTLLISNPTISQRLFTWADIVYAVFFGAAVITI

: : : :. . *: .. ::

ARQILFTGSVDSNKVVGSVSLFLLLGLMWTIIYLLVMEFSPEAFTGMTAAPWVENFSRMA

ARQILFTGHVNSNKVIGSVALFLLLGLMWAIAYLILLEFSPEAFTGMEAISWGQNFSNAA

VRKILFSGQIDTNKLIGSVALFLLLGLMWAVAYLLLLELDPFAFRGLEAIPWEDNFSNSA

GSRISHEKEVNLNVVRGGVCIYLLLGLLWFFLYKIIIFFDVSAFSFPENISKDS----LF

SQQLNKVQKVDQNALLGAISVYLLIGVFWFLLYRISYIISPTNFNELQSDGINN--FILL

:: :: * : *.:.::**:*::* . * : :. * .

YFSFVTLT**TLGYGD**ISPLSPFAQVVVYLEAIAGVFYMAIVVASLVSS---SQSNQDK---

YFSFVTLT**TLGYGD**ISPLTPLAQVVVYLEAITGVFYMAIVVSSLVSSNIDHQVNKNG---

YFSFVTLT**TLGYGD**ISPVTPIAKTLVYLESVVGVFYMAVVVSSLVSSNLGRNTPR-----

YFSFTTLT**TLGYGD**ILPKNAFAMTFANAEALVGQIYPAVVIAKLVSLYEHK---------

YFSFTTLT**TLGYGD**ITPTDSIAMGLSNMEAIVGQMYSVIFVARLVSLYTTDLNCHSQIRE

****.********** * .:* . *::.* :* .:.:: ***

-----

-----

-----

-----

EQESD
