# Supplementary figures and images for: A Novel Potassium Channel in Photosynthetic Cyanobacteria
Source: PLoS One. 2010 Apr 12;5(4):e10118. doi: 10.1371/journal.pone.0010118 (PMC2853561; doi:10.1371/journal.pone.0010118)

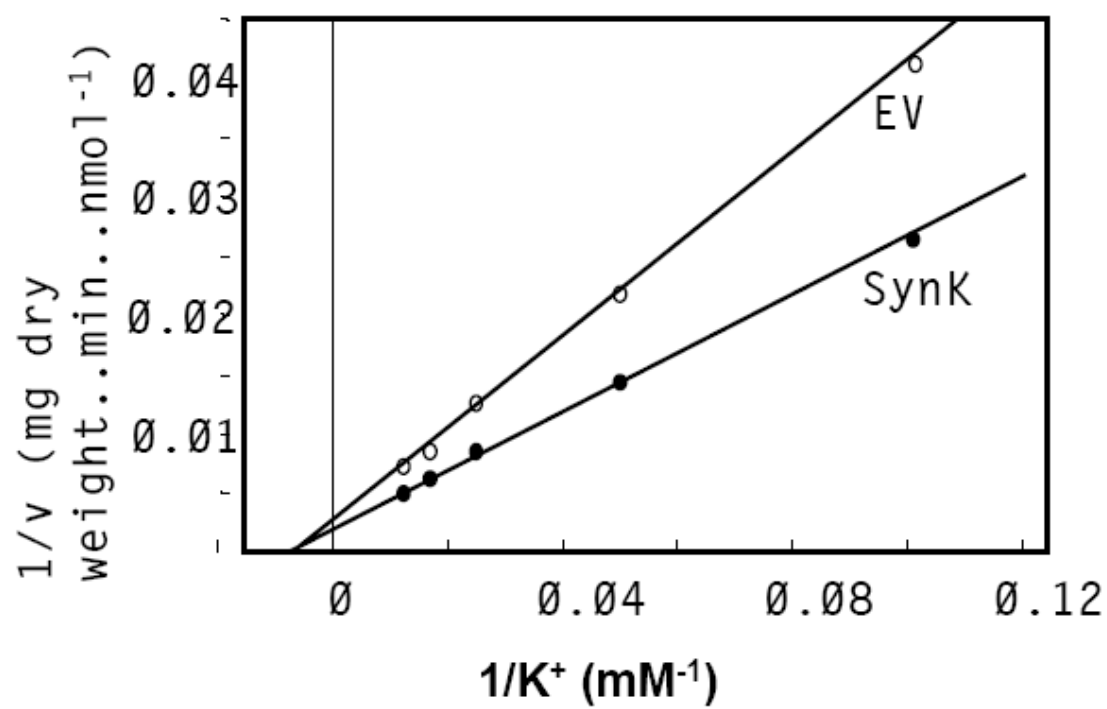

Supplement: Figure S2 — Potassium uptake by K+-depleted E.coli containing SynK or empty vector. Net potassium uptake measurements by K+-depleted E. coli cells in the presence of 10 to 80 mM KCl revealed Vmax values of 553 and 460 nmol min−1 g−1 dry weight for SynK-expressing cells and for the control cells, respectively Lineweaver-Burk plot of K+ uptake data obtained from four independent experiments is shown. (0.02 MB PDF) [file pone.0010118.s003.pdf]

WT SynK

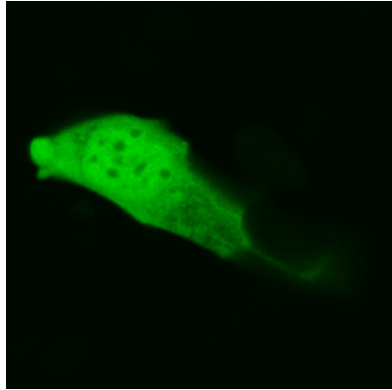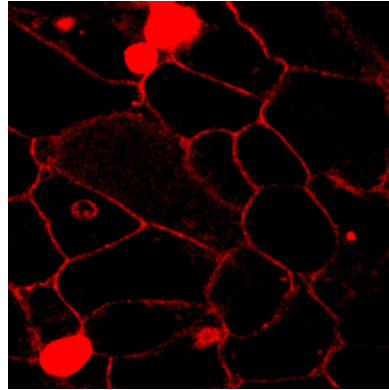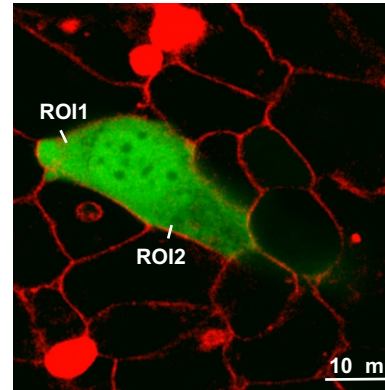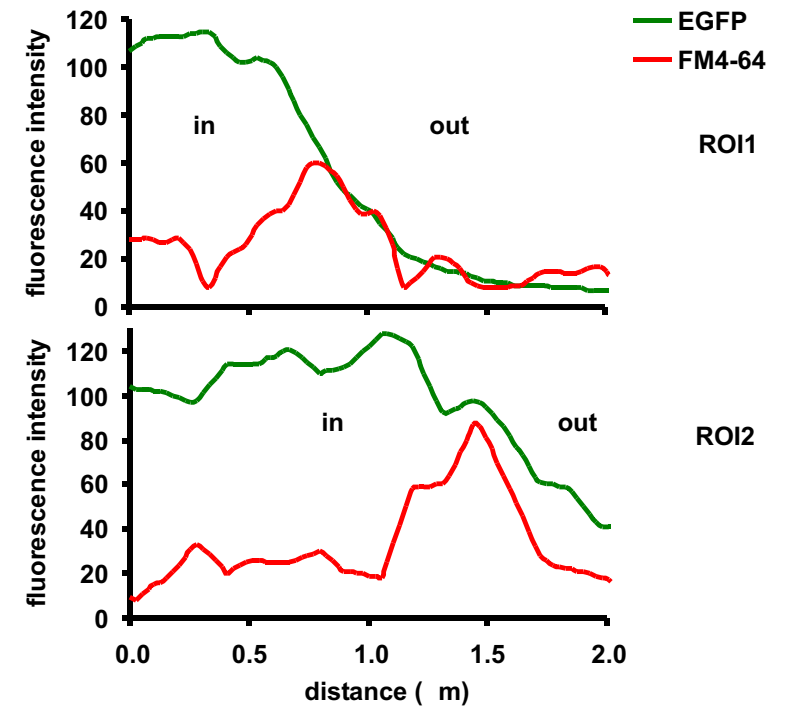

Mutant SynK

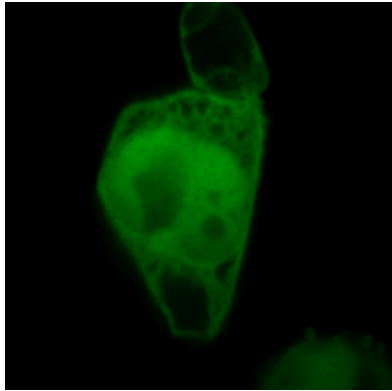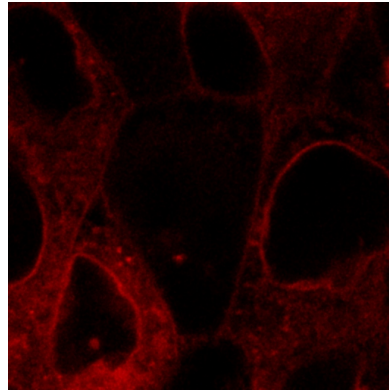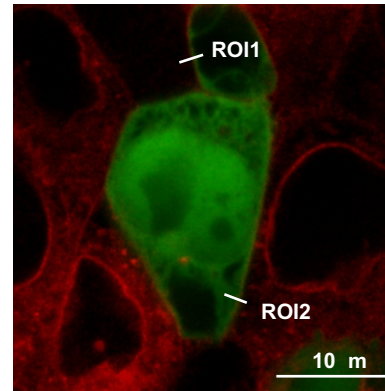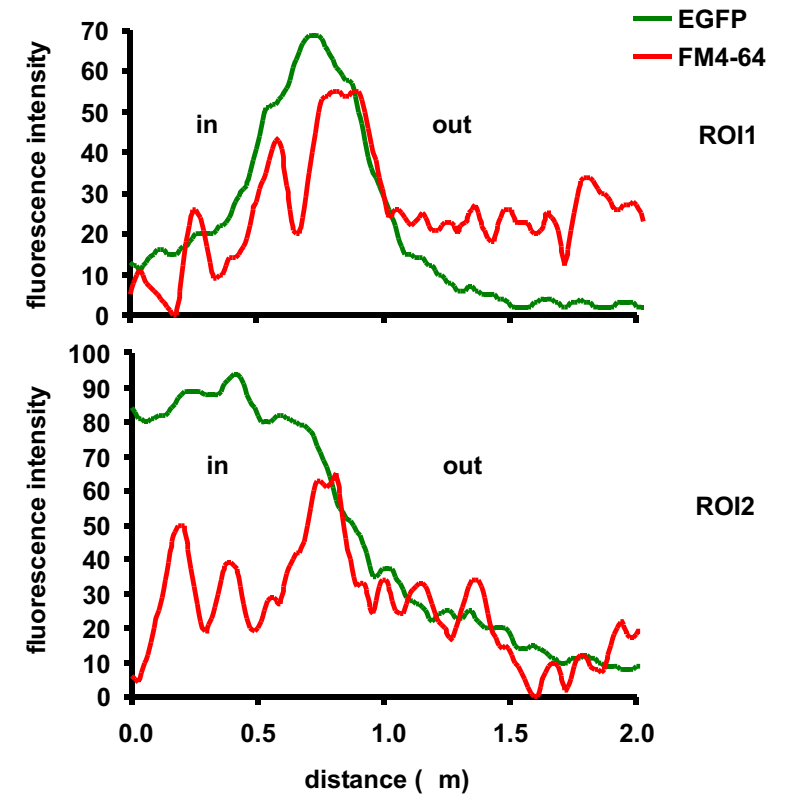

Supplement: Figure S3 — Expression of SynK and SynK mutant in Chinese Hamster Ovary cells. SynK-EGFP WT and mutant (non-conducting mutant with GAGD instead of GYGD in the pore region) fusion protein expression in CHO cell plasma membrane was revealed by confocal microscopy. Images with GFP fusion proteins (left images) and FM4-64 dye (central images) and merged signals (right images) are shown for WT SynK-GFP (upper panels) and mutant SynK-GFP (lower panels). Graphics shown beside the merged images represent profile plots of GFP (green) and FM4-64 (red) fluorescence intensity as a function of the distance for a particular region of interest (ROI), from inside the cell (in) to outside (out). Peaks falling in the same region correspond to co-localization. (0.48 MB PDF) [file pone.0010118.s004.pdf]

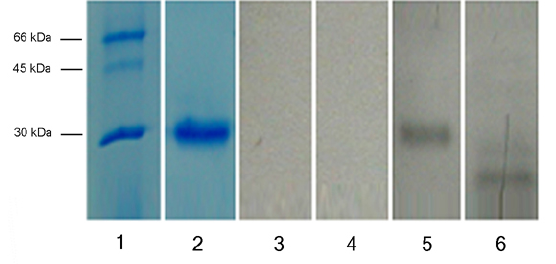

Supplement: Figure S4 — Anti-SynK antibody recognizes recombinant and native SynK. Recombinant protein (144 N-terminal amino acids of SynK fused with a 6 His-tag at C-terminus) was expressed in E. coli and purified as described in Materials and Methods. Protein was purified as a 30-kDa dimer (see lane 2). 30-kDa protein, recognized by anti-His antibody (not shown), was used for antibody production. Pre-immune antiserum did not recognize either purified 30 kDa protein (lane 3) or proteins in cyanobacteria whole-cell lysate (lane 4); serum from immunized rabbit clearly reacted with the recombinant protein (lane 5) and recognized SynK of 26 kDa in whole-cell lysate (in cells containing 0.1 µg chlorophyll) even at 1∶5000 dilution (lane 6). (0.16 MB DOC) [file pone.0010118.s005.doc]

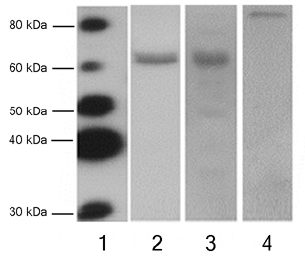

Supplement: Figure S5 — Anti-KPORE antibody recognizes other potassium channels. Anti-KPORE antibody was used at 1∶10000 dilution on whole-cell lysate of Jurkat lymphocytes, known to express Kv1.3 channel with apparent MW of 65 kDa (Magic Marks loaded on lane 1). Same bands were recognized by anti-KPORE (lane 2) and by a specific antibody against Kv1.3 (1∶200) (lane 3) in SDS-PAGE with 6 M urea. 50 µg total proteins were loaded. Anti-KPORE antibody also recognized purified GST-Kv1.3 protein (lane 4, 10 µg loaded, predicted MW 87 kDa) (production of GST-Kv1.3 is described in Gulbins et al, Biochim. Biophys. Acta, in press). Anti-KPORE antibody also recognized KCa3.1 in HCT116 colon cancer cell line (not shown), and monomeric as well as multimeric forms of the purified Kcv viral potassium channel (not shown) and of purified KvAP (kindly provided by P.Facci, not shown). (0.07 MB DOC) [file pone.0010118.s006.doc]

Figure S6.

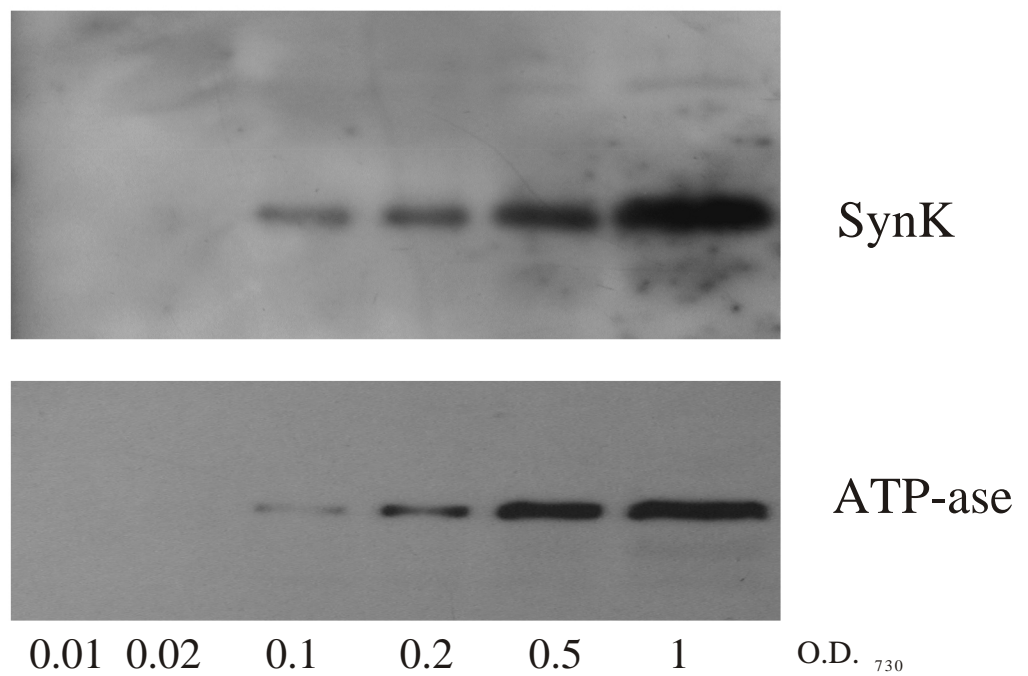

Supplement: Figure S6 — Anti-SynK antibody efficiently recognizes SynK in whole-cell lysate of cyanobacteria. Cells corresponding to the O.D. (at 730 nm) shown on the figure were solubilized in SB and loaded on SDS-PAGE. The blot was first developed with anti-SynK antibody and after re-stripping with anti-ATP-ase antibody (Agrisera). Efficiency of anti-Synk and anti-ATP-ase antibodies is comparable. (3.76 MB PDF) [file pone.0010118.s007.pdf]

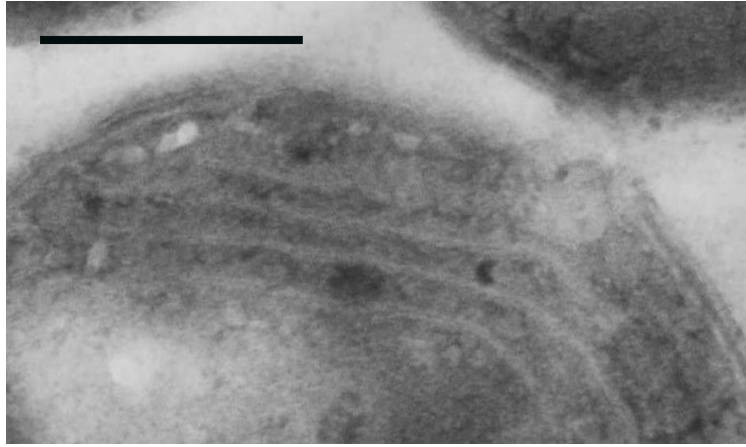

Supplement: Figure S7 — Secondary antibody does not label cyanobacteria in immunogold electron microscopy. As control, only secondary IgG was used. Bar: 500 nm. (0.05 MB PDF) [file pone.0010118.s008.pdf]

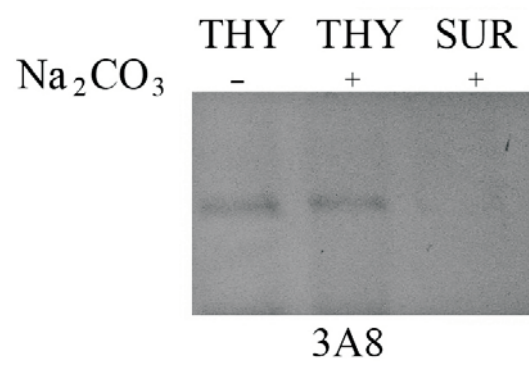

Supplement: Figure S9 — The 54 kDa protein is an integral membrane protein. Thylakoids (100 mg total proteins) were subjected to alkaline extraction (0.2 M Na2CO3 for 30 minutes), pelleted and both pellet and supernatants were loaded. The 54 kDa band is not present in the supernatant fraction indicating that it is an integral membrane protein. Blots were developed with the indicated antibodies. (0.10 MB PDF) [file pone.0010118.s010.pdf]

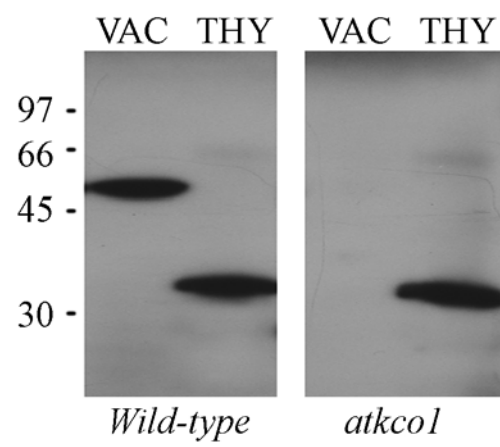

Supplement: Figure S10 — TPK1 locates to tonoplast in Arabidopsis. A specific monclonal antibody was used to reveal location of TPK1 in WT and atkco1 plants. Cells were fractionated and loaded on continuous sucrose gradient. Fractions positive for tonoplast TIP1 (VAC) or for thylakoid membrane D2 (THYL) were loaded. TPK1 is visible only in the vacuolar fraction of WT cells (at 50 kDa). An aspecific recognition is seen at approx. 35 kDa in thylakoids in both WT and mutant organisms. (0.06 MB PDF) [file pone.0010118.s011.pdf]
