# Supplementary material for: A Novel Potassium Channel in Photosynthetic Cyanobacteria
Source: PLoS One. 2010 Apr 12;5(4):e10118. doi: 10.1371/journal.pone.0010118 (PMC2853561; doi:10.1371/journal.pone.0010118)
Supplement: Figure S8 — Sequence homology between cyanobacterial SynK and Arabidopsis TPK3 (At4g18160). Aminoacid sequence alignments obtained by T-COFFEE algorithm. “*” - identical residues in all aligned sequences; “:” - conserved, “.” - semi-conserved substitutions. (0.21 MB PDF) [file pone.0010118.s009.pdf]

```
T-COFFEE, Version_7.71Mon Feb 23 21:41:54 WEST 2009
  Cedric Notredame
  CPU TIME:0 sec.
  SCORE=63
  *
   BAD AVG GOOD
  *
  gi|1001732|dbj|  :  63
  At4g18160        :  63
  cons             :  63

  gi|1001732|dbj SynK ---------------------------------------------
  At4g18160      TPK3 MANEGSDPLLQYMISPRLKKPPQLLFPLPEDNEVAIPMPMTPSEF

  cons


  gi|1001732|dbj|     ----MFGKYRQKNLDNWHYRNLFWSIVLLLFFTMFVKTRMGGTIT
  At4g18160           KERLIFGPFSCSPRDSSHFIDSMKQPS------------PSSSST

  cons                    :** :  .  *. *: : : .               ..: *


  gi|1001732|dbj|     SILFTVTILVMVKN-MAISSLWK----------------------
  At4g18160           ----------AVNNPFSDSSTLDPLLPPPPPQPEPWLSDQTSSHC

  cons                           *:* :: **  .


  gi|1001732|dbj|     ---TFLR--GLVAIALGCDLLT-LLISNPTISQRLFTWADIVYAV
  At4g18160           QGHALHRSKTAPAMAVINDLHHPIRQKDPTETSRS----------

  cons                   :: *     *:*:  **   :  .:** :.*


  gi|1001732|dbj|     FFGAAVITISQQLNKVQKVDQNALLGAISVYLLIGVFWFLLYRIS
  At4g18160           ------------------VVR-QAFALLVVYLSLGVLIYWLNRDH

  cons                                  * :   :. : *** :**: : * *


  gi|1001732|dbj|     YIISPTNFNELQSDGINNFILLYFSFTTLTTLGYGDITPTDSIAM
  At4g18160           YVVNQTHP---VVDG------LYFCIVTMCTIGYGDITPNSVVTK

  cons                *::. *:      **      ***.:.*: *:*******.. ::


  gi|1001732|dbj|     GLSNMEAIVGQMYSVIFVARLVSLYTTDLNCHS------------
  At4g18160           LFSIMFVLVGFGFIDILLSGMVS-YVLDLQESYMLDSAKRRDEPE

  cons                 :* * .:**  :  *::: :** *. **:


  gi|1001732|dbj|     ---------------------------------------------
  At4g18160           KRRSYIIDVKKGRMRIRLKVALALGVVVLCIAVGVGIMHFIEEIG

  cons
```

```
gi|1001732|dbj|    ---------------------------------------------
At4g18160          WLDSFYLSVMSVTTVGYGDRAFKTLPGRLFAAIWLLVSTLAVARA

cons


gi|1001732|dbj|    ---------------------------------------------
At4g18160          FLYLAEARVDKRNRERAKKVLCETMSVSQFFAADIDNNGCVSKAE

cons


gi|1001732|dbj|    --------------------------------------QIREEQE
At4g18160          YVIYKLKEMEKITDKDILPISKQFDKLDRCSNGKITLLDLLEGGS

cons                                                     :: *  .


gi|1001732|dbj|    SD
At4g18160          GD

cons               .*
```
