# Supplementary material for: A Novel Potassium Channel in Photosynthetic Cyanobacteria
Source: PLoS One. 2010 Apr 12;5(4):e10118. doi: 10.1371/journal.pone.0010118 (PMC2853561; doi:10.1371/journal.pone.0010118)
Supplement: Figure S11 — Pore region and YF residues are highly conserved between SynK and TPK channels of Arabidopsis. Voltage-gated Kv and KCNQ channels are characterized by a conserved pore region feature, namely, the presence of two tryptophans in tandem (W67 and W68 in KcsA) (Minor DL (2001) Potassium channels: life in the post-structural world. Current Opinion in Structural Biology, 11: 408–414). In plant shaker-like inward rectifier channels, the second tryptophan is highly conserved and the first is replaced by a tyrosine. These same positions are strongly conserved within other families of potassium channels, however, as different residues. Animal Kir channels harbour LF or SF residues in the same position (Minor 2001). Instead, in animal two-pore channels, in viral Kcv as well as in all plant two-pore channels the same positions are occupied by tyrosine and phenylalanine (YF). SynK has the same YF aminoacids in the corresponding position, further suggesting that SynK might have given origin to two-pore channels during evolution. Interestingly, GORK and SKOR outwardly rectifying voltage-dependent channels, also harbour YF residues in the corresponding position but, in contrast to TPK3, do not show significant homology with SynK. Aminoacid sequence alignments obtained by T-COFFEE algorithm. “*” - identical residues in all aligned sequences; “:” - conserved, “.” - semi-conserved substitutions. YF residues, typical of Kcv, animal and plant two-pore potassium channels are indicated. At4g01840: TPK5; Atg1g02510: TPK4; At4g18160: TPK3; At5g46370: TPK2; At5g55630: TPK1. (0.03 MB PDF) [file pone.0010118.s012.pdf]

```
At4g01840    HYSGIETHP-------V--VDALYFCIVTMCTIGYGDIAPLTPWTKIFAVV
At1g02510    QFSGTETNL-------F--VDAFYFSIVTFSTVGYGDIVPSTSTTKILTIV
At4g18160    HYVVNQTHP-------V--VDGLYFCIVTMCTIGYGDITPNSVVTKLFSIM
At5g46370    SYNVKQTHP-------V--VDALYFCIVTMCTIGYGDITPDSVVTKLFSIF
At5g55630    QISGHKTSG-------V--VDALYFCIVTMTTVGYGDLVPNSSASRLLACA
SynK         SYIISPTNFNELQSDGINNFILLYFSFTTLTTLGYGDITPTDSIAMGLSNM

cons               *         .  .  :**.:.*: *:****:.*    :  ::
```
